# Nasopharyngeal Carcinoma Subtype Discovery via Immune Cell Scores from Tumor Microenvironment

**DOI:** 10.1155/2023/2242577

**Published:** 2023-03-31

**Authors:** Yanbo Sun, Yun Liu, Hanqi Chu

**Affiliations:** Department of Otolaryngology-Head and Neck Surgery, Tongji Hospital, Tongji Medical College, Huazhong University of Science and Technology, 1095 Jiefang Avenue, Wuhan 430030, China

## Abstract

**Background:**

Nasopharyngeal carcinoma (NPC) is one of the most prevalent cancers with a poor prognosis. Immunotherapy, especially immune checkpoint blockade (ICB), is becoming a potential therapeutic choice for NPC patients. Thus, the identification of patients who could benefit from immunotherapy is clinically significant.

**Methods:**

The NPC expression profiles from GSE102349 were used to calculate the cell scores of the tumor microenvironment (TME). The consensus clustering method was utilized to identify the potential molecular subtypes among NPC samples. The hub genes were selected from subtype-specific genes by bioinformatics analysis. Machine learning models, including random forest (RF) and support vector machine (SVM) algorithms, were constructed to predict the immune subtype.

**Results:**

In the present study, we identified two TME subtypes among NPC patients. Patients with the S1 subtype have higher levels of immune cells, immune checkpoint genes, and prognosis. Using expression data profiles of NPC patients, we constructed machine learning models for predicting TME subtypes of NPC patients. This model consists of 8 genes (LCK, CD247, FYN, ZAP70, SH2D1A, CD3D, CD3E, and CD3G). Among them, LCK, FYN, SH2D1A, and CD3D were associated with better prognoses. Among the two constructed models, SVM exhibited a higher area under curve (AUC) of 0.977, when compared with RF (AUC = 0.966). The web server based on the constructed machine learning models will contribute to the identification of NPC patients likely to benefit from ICB therapies.

**Conclusions:**

This study identified NPC subtypes and provided an accurate model to select individuals who are most likely to respond to ICB.

## 1. Introduction

Nasopharyngeal carcinoma (NPC) is a rare cancer in most regions of the world, accounting for only 0.7% of all cancers [1]. In 2020, more than 130,000 new cases of NPC were recorded globally, along with approximately 80,000 fatalities [2]. It is important to note that the geographical distribution of NPC samples is very unbalanced, with over 70 percent of NPC samples reported in Southeast Asia [3]. The peak incidence of NPC is 50 to 60 years of age, and males have a higher incidence of NPC than their female counterparts [4]. The etiologic factors of NPC include EBV infections, genetic factors, and environmental factors [4]. The preferred options for the treatment of NPC are primarily comprised of chemoradiotherapy and adjuvant chemotherapy [5]. The clinical outcome for individuals suffering from NPC is unfavorable due to tumor relapse and early migration [6]. For example, the median overall survival (OS) for patients with distant metastasis was only 15.6 months [7]. Thus, studies to identify novel and effective treatment therapies for NPC are urgently required.

One strategy to improve the OS of NPC patients with distant metastasis is to combine immune-checkpoint blockade (ICB) and chemotherapy. More than 90 percent of patients showed overall response to the combination of PD1 antibody with chemotherapy [8]. In another phase II study that aimed to investigate the efficacy of PD-1 antibody in metastatic NPC patients who had progressed after chemotherapy, the objective response rate (ORR) was 29.7% [9]. KEYNOTE-028 (NCT02054806) study is a phase I trial of PD1 antibody in NPC patients. A total of 7 out of 27 patients (ORR 26%) experienced an objective response to PD1 antibody [10]. However, there are some potential challenges for ICB treatment. First, in various solid tumors, only a small percentage of patients could benefit from long-term treatment of ICB (about 20 percent) [11]. Moreover, in NPC patients treated by ICB, severe drug side effects were found among 87 percent of patients [8]. Besides, the cost of ICB is also very high. Thus, the identification of NPC patients deriving a benefit from anti-PD1 agents is needed. Multiple biomarkers for ICB treatment have been recognized. For instance, earlier research has explored tumor mutation burden (TMB) [12], CD8+ T cells [13], and PDL1 expression [14]. TMB, on the other hand, has the drawback of having a cutoff value that changes according to the kind of tumor [15]. Additionally, regulatory T lymphocytes may decrease CD8+ T cell activity, and PDL1 expression confronts the challenge of intratumor and tumor site variation [16]. As a result, no reliable ICB treatment biomarker has been identified.

NPC, similar to many tumors, is an illness characterized by intratumoral heterogeneity. Thus, it is indeed vital to divide them into distinct subtypes that have different molecular features, specific therapies, and clinical outcomes. On the other hand, TME that contains immune cells, fibroblasts, and extracellular matrix is a crucial element of tumors, and it plays a crucial role in tumor development, migration, therapeutic sensitivity, and relapse [17]. Therefore, it is possible to divide NPC patients into multiple subtypes by the diversity and intricacy of the TME.

In the present study, we identified two TME subtypes among NPC patients. Patients with the S1 subtype have higher levels of immune cells, immune checkpoint genes, and prognosis. Using expression data profiles of NPC patients, we constructed machine learning models for predicting TME subtypes of NPC patients. This model consists of 8 genes (LCK, CD247, FYN, ZAP70, SH2D1A, CD3D, CD3E, and CD3G). Among them, LCK, FYN, SH2D1A, and CD3D were associated with better prognoses. The web server based on the constructed machine learning models will contribute to the identification of NPC patients deriving benefit from ICB therapies.

## 2. Materials and Methods

### 2.1. Data Resources

Three NPC gene expression data sets, including GSE12452 [18], GSE68799, and GSE102349 [19], were downloaded from GEO. GSE68799 contained 42 NPC tissue samples, GSE12452 contained 31 NPC samples, and GSE102349 contained 113 NPC samples. The clinical parameters of these three studies are shown in Supplementary Table 1. The validation dataset for evaluating TME subtypes with ICB response included IMvigor210 [20], GSE35640 [21], GSE78220 [22], and GSE111636 studies. IMvigor210 study contains 195 bladder cancer samples (PD-L1 antibody), GSE35640 study contains 65 lung cancer samples (MAGE-A3 immunotherapy), GSE78220 contains 28 melanoma samples (PD-1 antibody), and GSE111636 contains 11 advanced urothelial tumors (PD-1 antibody). The gene expression matrix and clinical information of datasets were collected. Ethical approval was not necessary for this study because our study is a bioinformatic analysis.

### 2.2. TME Cell Scores

In order to calculate the scores of TME cells, ssGSEA, MCP-counter, and ESTIMATE were used. The ssGSEA algorithm is an extension of the GSEA method and could compute an aggregated enrichment score for a gene set. Based on a list of immune metagenes, scores of 28 kinds of immune cells were calculated by ssGSEA [23]. The MCP-counter is a method that can evaluate the values of 8 immune and 2 stromal cells [24]. ESTIMATE is a method that can infer the immune, stromal, tumor, and ESTIMATE scores (the sum of immune and stromal cells) [25]. There are several reasons for selecting these methods. (1) The cell scores calculated by these methods could be compared between samples. (2) The combination of these methods contains the main cell types in TME (immune cells were mainly calculated from ssGSEA, endothelial cells and fibroblast were calculated from MCP-counter, and tumor cells were calculated from ESTIMATE). (3) These methods were the most prevalent methods for quantification of the absolute abundance of cells in TME. (4) These methods are available as R packages.

### 2.3. Consensus Clustering (CC) Analysis

A total of 42 TME cell scores were chosen for the CC analysis using the R ConsensusClusterPlus package [26]. And the most appropriate TME subtype numbers were selected by the plot of relative change in area under CDF curve, the plot of average silhouette width, the plot of tracking plot, the plot of consensus score matrix, and the plot of TSNE results. The log-rank test was used to assess the discrepancy in progression-free survival (PFS) across two different subtypes.

### 2.4. Identification of Differentially Expressed Genes and Enriched Pathways

The “limma” R package was used to identify DEGs among NPC subtypes [27]. A ∣log2FoldChange | >0.8 and a *p* value < 0.05 were used to identify DEGs. Based on GSEA, biological process (BP), cellular component (CC), molecular function (MF), Kyoto Encyclopedia of Genes and Genomes (KEGG), and Reactome were applied to identify functional pathways of NPC subtypes by log2FoldChange values. GSEA analysis was conducted by R fgsea package. This package implements a revolutionary approach that effectively reuses a single sample several times, hence, accelerating the analysis. This package enables the rapid generation of millions of permutations in a matter of minutes, resulting in very precise *p* values. Enriched items with the *p* value < 0.05 were considered statistically significant.

### 2.5. Weighted Correlation Network Analysis (WGCNA)

In the process of WGCNA, the outliers were identified and removed by WGCNA package [28]. Then, *β* values and scale free *R*2 were adjusted to form a scale-free coexpression network. After that, genes with higher connections were clustered to construct modules. In our study, modules were generated by the parameters “minModuleSize = 10” and “mergeCutHeight = 0.15.” The relationships of modules with clinical characteristics of patients were calculated, and the module having the greatest correlation value with NPC subtypes was chosen.

### 2.6. Protein-Protein Interaction (PPI) Network Creation

Genes from the chosen module were used to build the PPI network based on the STRING database. STRING database contains PPIs from sources of experiments and assists in the identification of key regulator genes. The inclusion criteria for protein interactions in the String database were set as “confidence > 0.4”. Then, the PPIs were uploaded to Cytoscape software to construct the PPI network. The genes with the highest degree value were selected as hub genes.

### 2.7. The Construction and Validation of Machine Learning Models

RF and SVM algorithms from the “caret” package [29] were used to construct the NPC subtype prediction models. The mRNA expression levels of hub genes were needed in the model training phase. (1) Training (50%) and testing data (50%) were split from NPC samples of GSE102349. (2) Fivefold cross-validation was selected to determine the optimal tuning parameters. (3) AUC values in the testing dataset of the constructed models were calculated to evaluate their prediction ability. ICB datasets (IMvigor210, GSE35640, GSE78220, and GSE111636) were selected in the investigation of the association of NPC subtypes with ICB efficacy.

### 2.8. NPC Subtype Prediction Web Server

The SVM model, built for NPC subtype prediction, was used to develop a web server. The web server was provided by the R language “shiny” package [30]. The web server can be accessed with any computer system and web browser.

## 3. Results

### 3.1. Construction of Molecular Subtypes Based on Cell Scores

**Figure 1:**
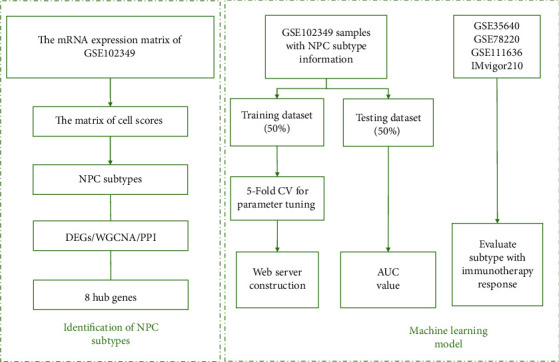
The flowchart of this study.

**Figure 2:**
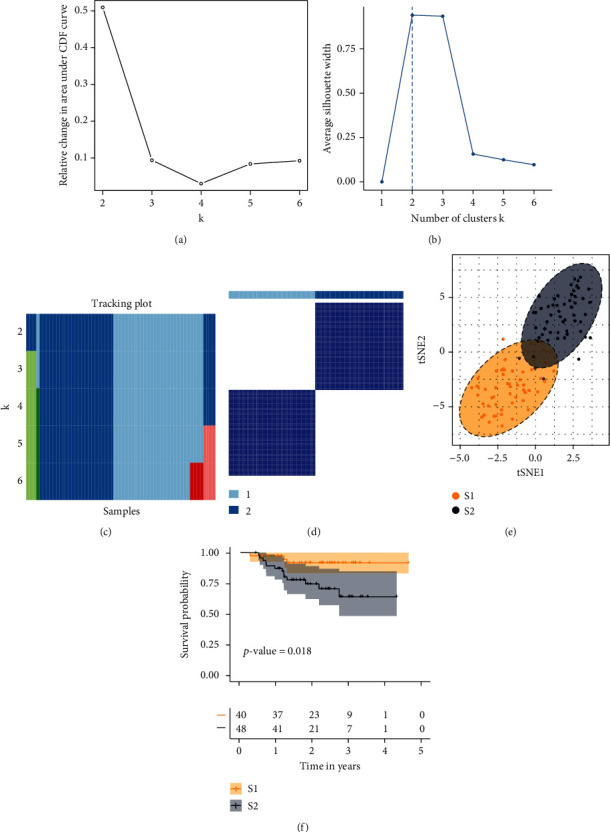
Consensus clustering for NPC samples from GSE102349. (a) The relative change in area under CDF curve for each k subtype. (b) Silhouette width of each subtype in NPC. The best subtype number was should be the k value with the highest value of average silhouette width. (c) In the tracking plot, the percentages of subtypes were indicated by different colors. (d) Consensus matrix heat map plots when *k* = 2. (e) t-SNE-plot for RNA-sequencing data from NPC samples from GSE102349. (f) Five-year Kaplan-Meier curves for progression-free survival of NPC patients stratified by the NPC subtypes. CDF: consensus clustering cumulative distribution function.

The flowchart of this study is shown in Figure 1. The scores of 42 cell types for NPC samples, calculated by ssGSEA, MCP, and ESTIMATE methods, were used to study NPC subtypes in GSE102349. ConsensusClusterPlus was adopted to split NPC tumors into *k* subtypes (*k* = 2–6). Based on the plot of relative change in area under the CDF curve, *k* = 2 was optimal (Figure 2(a)). Based on the plot of average silhouette width, *k* = 2 was optimal (Figure 2(b)). Based on the tracking plot, *k* = 2 was optimal (Figure 2(c)). The plot of the consensus score matrix (*k* = 2) for NPC samples was plotted (Figure 2(d)). In addition, TSNE results showed that there are two main subtypes among NPC patients (Figure 2(e)), referred to as S1 and S2. In general, S1 showed a better overall prognosis than S2 (Figure 2(f)).

### 3.2. Differences in Immune Cell Infiltration of Different Subtypes

**Figure 3:**
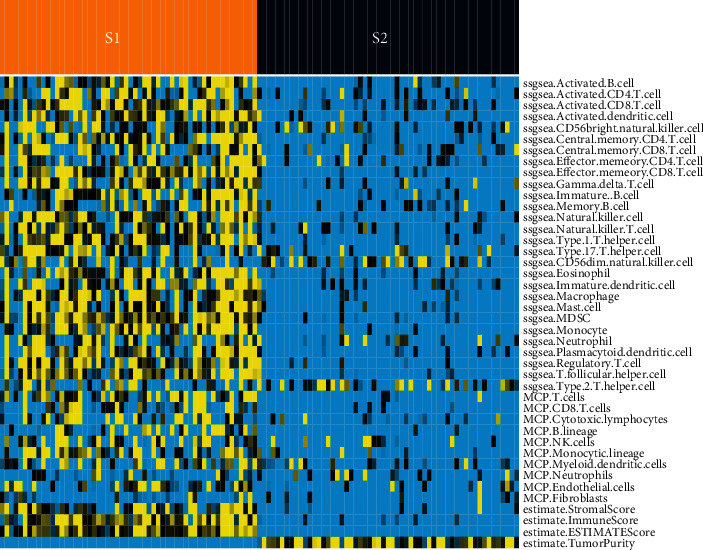
Cell scores in two different NPC subtypes are illustrated by the heat map (yellow: higher value; blue: lower value).

**Figure 4:**
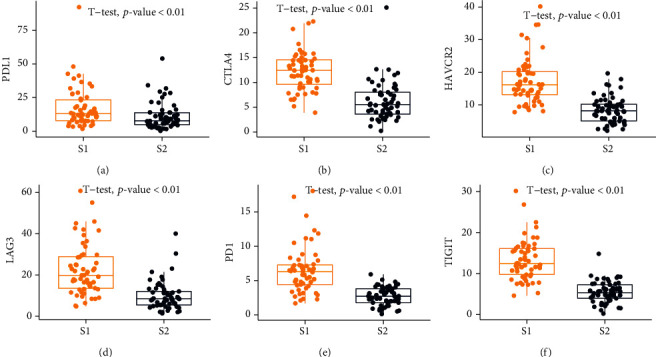
Differential expression of the immune checkpoint genes PDL1, CTLA4, HAVCR2, LAG3, PD1, and TIGIT among two NPC subtypes, as evaluated by *t*-test.

Among the two subtypes, S1 had a higher degree of immune cell infiltration than S2. A “desert”-like characteristic was seen in S2, which was devoid of T cells, particularly CD8 T cells, in the TME (Figure 3). However, S2 demonstrated higher tumor purity than S1. We also investigated the expression values of immune checkpoint genes (i.e., PD1, PDL1, and CTLA4) that are associated with immune escape. The expression levels of genes are higher in the S1 subtype (Figure 4). Furthermore, we compared immune subtypes with tumor stage and TMB, and we observed no statistically significant differences between NPC subtypes (Supplementary Table 2).

### 3.3. Validation of Molecular Subtypes on Two Independent Datasets

In the independent dataset GSE12452, the same R package ConsensusClusterPlus was used to identify the potential subtypes of NPC samples. Based on the consensus matrix plot, relative change in area under the CDF curve, and the plot of tracking plot, the plot of average silhouette width, *k* = 2 was optimal (Supplementary Figure 1A-1D). The cell scores for two NPC subtype samples were plotted (Supplementary Figure 2). In another independent dataset, GSE68799, two subtypes were also found (Supplementary Figure 3A-3D). The cell scores for two NPC subtype samples were also plotted (Supplementary Figure 4).

### 3.4. Identification of Subtype-Relevant DEGs

With the use of the “limma” package, we obtained 1072 DEGs that were differently expressed between S1 and S2 subtypes. Compared to the S1 subtype, 155 (15%) genes were upregulated, while 917 (85%) genes were downregulated in the S2 subtype samples. For the DEGs, a volcano plot is constructed and shown (Supplementary Figure 5).

### 3.5. Functional Enrichment Analysis

In the terms of the biological process (Supplementary Table 3), GSEA results indicated that upregulated genes in S2 were enriched in cardiac chamber development, DNA-dependent DNA replication maintenance of fidelity, circadian rhythm, and spinal cord development. Upregulated genes in S1 were enriched in cellular response to lipoprotein particle stimulus, positive regulation of kinase activity, regulation of T cell receptor signaling pathway, and movement in environment of other organism involved in symbiotic interaction. For the molecular function (Supplementary Table 4), the enriched terms of upregulated genes in S2 included Mannosyltransferase Activity, Trna Binding, Dna Secondary Structure Binding, and Magnesium Ion Binding. Upregulated genes in S1 included Lipopolysaccharide Binding, Amyloid Beta Binding, G Protein Coupled Chemoattractant Receptor Activity, and Peptide Receptor Activity. For the cellular component (Supplementary Table 5), the enriched terms of upregulated genes in S2 included Histone Deacetylase Complex, Ubiquitin Ligase Complex, and Nuclear Ubiquitin Ligase Complex. Upregulated genes in S1 included Cytoplasmic Ubiquitin Ligase Complex. As to KEGG (Supplementary Table 6), upregulated genes in S2 were mainly associated with the pathways of oxidative phosphorylation, pyrimidine metabolism, and lysine degradation. Upregulated genes in S1 were mainly associated with the pathways of tryptophan metabolism, other glycan degradation, and glycosaminoglycan degradation. Moreover, Reactome (Supplementary Table 7) showed that upregulated genes in S2 were mainly associated with Translation, RNA Pol III Transcription Initiation from Type 2 Promoter, and RNA Pol I Transcription Termination. Upregulated genes in S1 were mainly associated with signaling by Rho Gtpases, antigen processing cross presentation, Trif-mediated Tlr3 signaling, and endosomal vacuolar pathway.

### 3.6. Detection of Gene Coexpression Modules Correlated with NPC Subtypes

**Figure 5:**
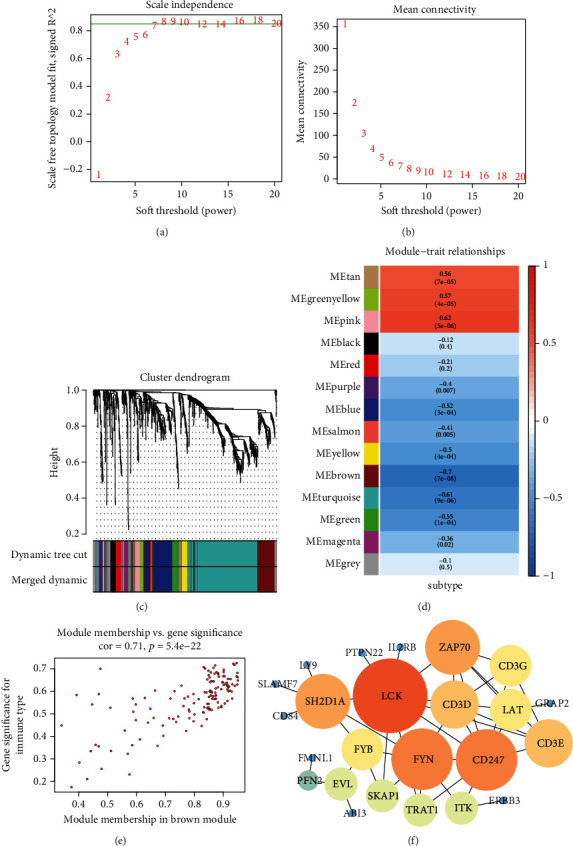
(a) The influence of power values on the scale independence. (b) The influence of power values on the average connectivity. (c) In the cluster dendrogram, each branch indicates one gene, and each color indicates a module. (d) Correlations between the gene modules and NPC subtypes. (e) Scatter diagram for module membership vs. gene significance in the brown module. (f) The genes from brown module were selected to construct the protein-protein interaction network.

1072 DEGs were used for WGCNA. The outlier samples were removed (Supplementary Figure 6), and the “softthreshold = 8” was chosen to build a scale-free network (Figures 5(a) and 5(b)). A total of 14 gene modules were discovered after setting the minimum cluster size as 10 (Figure 5(c)). The association of gene modules with the NPC subtype was then explored. We found that the brown module (*R* = −0.70, *p* value < 0.01) was significantly associated with the immune subtype of NPC (Figure 5(d)). In addition, the genes in the brown module demonstrated high module membership (MM) and gene significance (GS) (Figure 5(e)). The brown module was chosen for further investigation because it had the largest negative connection with NPC subtypes of all the modules tested.

### 3.7. Survival Analysis of Hub Genes

**Figure 6:**
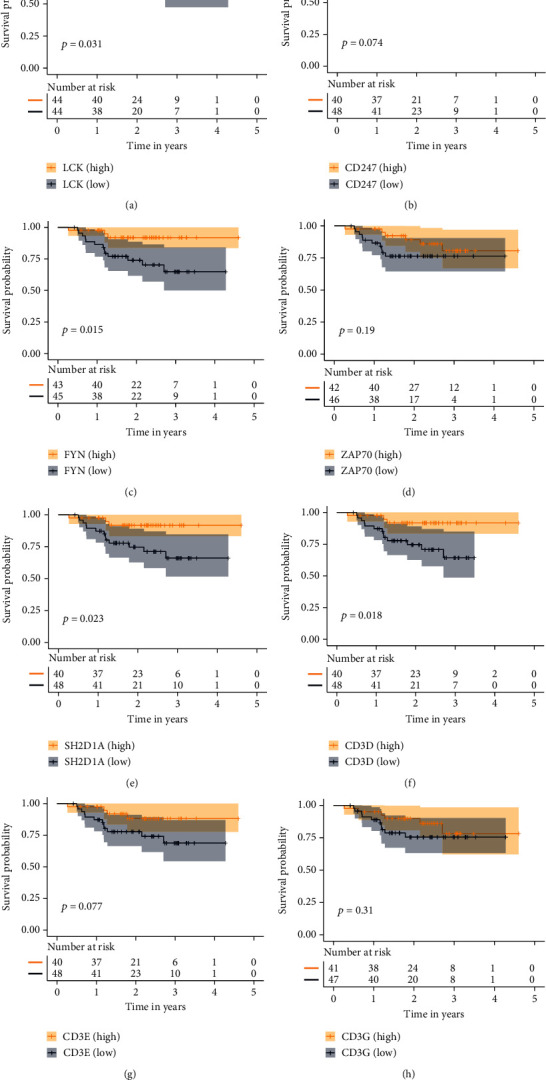
Hub-gene survival analysis. (a)–(h) Survival analysis of 8 hub genes divided according to the median value of their respective expression levels. *p* value < 0.05 was considered significant.

In the brown module, 8 hub genes (LCK, CD247, FYN, ZAP70, SH2D1A, CD3D, CD3E, and CD3G) were identified by the degree value in the protein-protein interaction network (Figure 5(f)). Based on median expression values, we compared the survival differences of high and low hub gene groups. Patients with a lower level of LCK (Figure 6(a)), FYN (Figure 6(c)), SH2D1A (Figure 6(e)), and CD3D (Figure 6(f)) exhibited significantly shorter PFS (*p* value < 0.05). Similar results were observed in CD247 (Figure 6(b)), ZAP70 (Figure 6(d)), CD3E (Figure 6(g)), and CD3G (Figure 6(h)).

### 3.8. Construction of Prediction Models of NPC Subtypes

**Figure 7:**
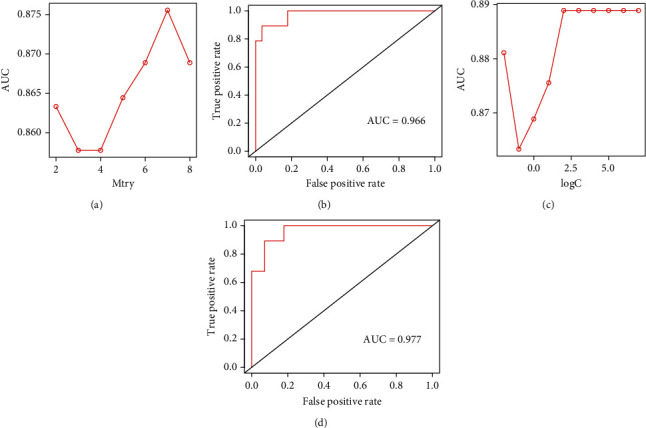
Construction and validation of machine learning models for predicting NPC subtypes. (a) Parameter optimization of RF model by 5-fold cross-validation in the training set. The mtry: number of variables randomly sampled as candidates at each split. (b) ROC curve analysis of the performance of RF in the test set. (c) Parameter optimization of SVM model by 5-fold cross-validation in the training set. C parameter in SVM is penalty parameter of the error term. (d) ROC curve analysis of the performance of RF in the test set.

The mRNA expression levels of genes (LCK, CD247, FYN, ZAP70, SH2D1A, CD3D, CD3E, and CD3G) from GSE102349 were selected to build RF and SVM models for NPC subtype prediction. The median value was used to translate mRNA expression values from integer data (0–1) to categorical values (“high” or “low”). The optimal parameters for the RF and SVM models were selected as “mtry = 7” and “*C* = 4” by the best AUC values (Figures 7(a) and 7(c)). After SVM and RF model construction, RF and SVM models scored satisfactorily, exhibiting AUC values of 0.966 and 0.977, respectively, in the testing dataset (Figures 7(b) and 7(d)).

### 3.9. Evaluation of the Correlation of NPC Subtype with ICB

**Figure 8:**
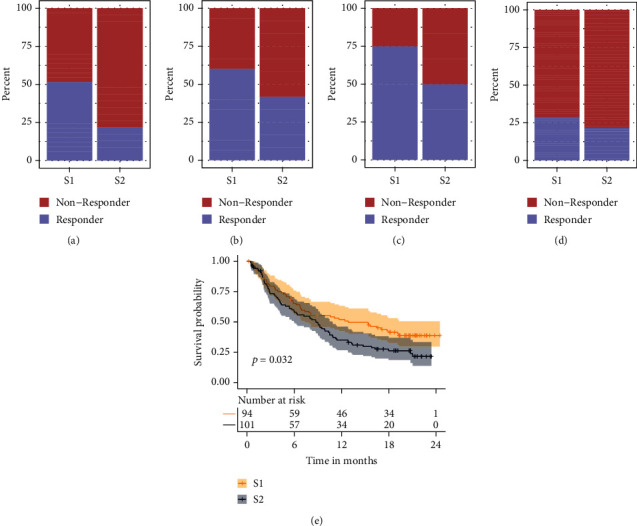
The correlation of NPC subtypes with response to ICB. (a) Analysis of response data of different subtype patients who had received ICB treatment (GSE35640). (b) Analysis of response data of different subtype patients who had received ICB treatment (GSE78220). (c) Analysis of response data of different subtype patients who had received ICB treatment (GSE111636). (d) Analysis of response data of different subtype patients who had received ICB treatment (IMvigor210). (e) Analysis of overall survival of different subtype patients who had received ICB treatment (IMvigor210).

IMvigor210, GSE35640, GSE78220, and GSE111636, containing the gene expression and ICB response data, were used to evaluate the correlation of NPC subtype with ICB. The subtypes of samples from these datasets were determined by the SVM model and expression values of LCK, CD247, FYN, ZAP70, SH2D1A, CD3D, CD3E, and CD3G. The ICB response rates of S1 in GSE35640, GSE78220, GSE111636, and IMvigor210 were 0.51 (Figure 8(a)), 0.60 (Figure 8(b)), 0.75 (Figure 8(c)), and 0.29 (Figure 8(d)). The ICB response rates of S2 in GSE35640 (Figure 8(a)), GSE78220 (Figure 8(b)), GSE111636 (Figure 8(c)), and IMvigor210 (Figure 8(d)) were 0.22, 0.41, 0.50, and 0.21. S1 patients were linked with better OS than S2 (Figure 8(e)).

### 3.10. Web Server Development

A web server with the name of Nasopharyngeal Carcinoma Subtype Prediction (NPCSP) via https://npcstudy.shinyapps.io/subtype/ was constructed for NPC subtype prediction. The expression values of eight genes (LCK, CD247, FYN, ZAP70, SH2D1A, CD3D, CD3E, and CD3G) are needed for prediction. Then, the NPC subtype will be predicted by expression values and the SVM model. The tutorial for using the constructed web server was provided in Supplementary Figure 7.

### 3.11. Discussion

Like many cancers, the intratumoral heterogeneity of NPC is the major reason for the significant distinct prognosis among NPC patients. Classifying the NPC subtypes and selecting the right therapeutic strategies are crucial. TME has a critical role in tumor development, migration, therapeutic tolerance, and disease relapse. Thus, by evaluating the TME, it is feasible to categorize NPC patients into several subgroups. In this study, we calculated the TME cell cores and then classified NPC samples into two subtypes: S1 and S2.

Among two NPC subtypes, S1 had the greater immune score, a greater stromal score, and a lower tumor purity than S2. Since S1 was enriched in T cells, the samples in the S1 subtype could be classified into “hot” tumors. Hot tumors were usually linked with greater T cells, immune checkpoints, and a better response to ICB. Thus, S1 NPC patients are prone to being responders to ICB because S1 and S2 are considered to be “hot-tumor” and “cold-tumor,” respectively. Results from independent datasets also confirmed that S1 patients have a greater probability of ICB response.

In the present work, we created an RF model to predict the NPC subtype by the expression values of eight genes. The model's AUC value suggested that it performed well in the testing dataset. We created an online web server to make this RF model accessible for researchers. Users only need to provide expression values of eight genes (LCK, CD247, FYN, ZAP70, SH2D1A, CD3D, CD3E, and CD3G) to the web server. Expression values and the SVM model will be used to predict the NPC subtype. Consequently, our work provides a suitable strategy for predicting the NPC subtype and therefore advising on the ICB therapy decision.

In the previous study, based on ligands and receptors, a model reached an AUC value greater than 0.7 in an independent validation dataset (GSE35640) for predicting immunotherapy response [31]. Their results also found that the AUC of PDL1 expression was 0.69, and the AUC of IFNG expression was 0.75. Another study provided a machine learning model based on the random forest algorithm and 11 genes to predict the immunotherapy response subgroups [32]. The model reached the AUC value of 0.76 in the testing dataset. In our study, the model of SVM had a higher AUC value than other studies, since the AUC value was 0.977.

The hub genes used in machine learning model construction are crucial in the immune signaling pathways. In T cells, LCK plays a critical role in the regulation of T cell receptor (TCR) signaling [33]. CD3–TCR complex, comprised of CD3D, CD3E, CD3G, and CD247, is the major regulator of T cell proliferation and stimulation [34, 35]. Fyn is a membrane proximal and nonreceptor tyrosine kinase and could initiate TCR and several natural killer cell activation receptors [36]. ZAP-70 is a cytoplasmic protein tyrosine kinase that is required for the antigen receptor to initiate T cell responses [37]. SH2D1A also plays a critical role in the immune system since it is required in the interaction of T cells and B cells [38].

There are some advantages to our study. (1) TME cell scores that are crucial for tumor development and ICB response were used to classify subtypes. (2) Two independent datasets validated the identified subtypes. (3) Two machine learning models were constructed in our study, and the SVM reached an AUC value of 0.97 in the testing dataset. (4) The correlation of subtypes with the ICB response rate was validated by independent datasets. (5) A web server was provided for researchers to use the machine learning model. There are some disadvantages to our study. (1) An independent cohort that comprises NPC patients who were treated with ICB should be employed to verify the link between ICB and the NPC subtype. (2) The cohort size was limited, which could have resulted in a high rate of false-positive results. (3) There was no experimental validation for this study. Genetic and experimental research with a bigger sample size is necessary to corroborate the expression pattern of eight hub genes in the future.

## 4. Conclusion

Based on the TME cell scores, we identified two subtypes (S1 and S2) among NPC patients. The S1 subtype has higher levels of immune cells, immune checkpoint genes, and prognosis. We constructed machine learning models for predicting TME subtypes of NPC patients based on 8 genes (LCK, CD247, FYN, ZAP70, SH2D1A, CD3D, CD3E, and CD3G). The web server based on the constructed machine learning models will contribute to the identification of NPC patients deriving benefit from ICB therapies. The machine learning and web server provided in our study could be a reference for the individualized treatment of NPC patients.

## Data Availability

The data presented in this study are available within the article materials.

## Supplementary Materials

Supplementary 1 Supplementary Figure 1: consensus clustering for NPC samples from GSE12452. (a) Consensus matrix heat map plots when *k* = 2. (b) The relative change in area under CDF curve for each *k* subtype. (c) In the tracking plot, the percentages of subtypes were indicated by different colors. (d) Silhouette width of each subtype in NPC. The best subtype number was should be the *k* value with the highest value of average silhouette width.

Supplementary 2 Supplementary Figure 2: cell scores in two different NPC subtypes are displayed by heat map (yellow: higher value; blue: lower value).

Supplementary 3 Supplementary Figure 3: consensus clustering for NPC samples from GSE68799. (a) Consensus matrix heat map plots when *k* = 2. (b) The relative change in area under CDF curve for each *k* subtype. (c) In the tracking plot, the percentages of subtypes were indicated by different colors. (d) Silhouette width of each subtype in NPC. The best subtype number was should be the *k* value with the highest value of average silhouette width.

Supplementary 4 Supplementary Figure 4: cell scores in two different NPC subtypes are displayed by heat map (yellow: higher value; blue: lower value).

Supplementary 5 Supplementary Figure 5: volcano plot showing the gene expression differences between NPC subtypes. Blue dots, downregulated genes in S2. Red dots, upregulated genes in S2.

Supplementary 6 Supplementary Figure 6: clustering of samples and removal of outliers.

Supplementary 7 Supplementary Figure 7: the tutorial for using the constructed web server.

Supplementary 8 Supplementary Table 1: the clinical parameters of datasets (GSE12452, GSE68799, and GSE102349). Supplementary Table 2: associations of NPC subtypes with clinical features in the GSE102349 cohort. Supplementary Table 3: enriched BP items between S1 and S2 subtypes from gene set enrichment analysis. Normalized enrichment score (NES). Positive NES value means that the pathway is enriched in S2 subtype. Supplementary Table 4: enriched MF items between S1 and S2 subtypes from gene set enrichment analysis. Normalized enrichment score (NES). Positive NES value means that the pathway is enriched in S2 subtype. Supplementary Table 5: enriched CC items between S1 and S2 subtypes from gene set enrichment analysis. Normalized enrichment score (NES). Positive NES value means that the pathway is enriched in S2 subtype. Supplementary Table 6: enriched KEGG pathways between S1 and S2 subtypes from gene set enrichment analysis. Normalized enrichment score (NES). Positive NES value means that the pathway is enriched in S2 subtype. Supplementary Table 7: enriched Reactome items between S1 and S2 subtypes from gene set enrichment analysis. Normalized enrichment score (NES). Positive NES value means that the pathway is enriched in S2 subtype.
