# Supplementary figures and images for: Nasopharyngeal Carcinoma Subtype Discovery via Immune Cell Scores from Tumor Microenvironment
Source: J Immunol Res. 2023 Mar 31;2023:2242577. doi: 10.1155/2023/2242577 (PMC10234372; doi:10.1155/2023/2242577)

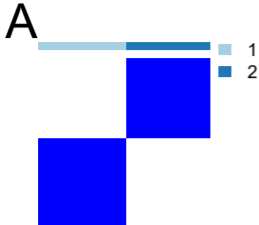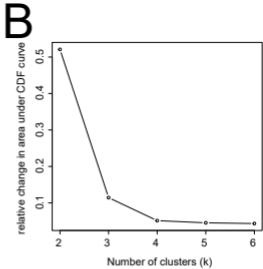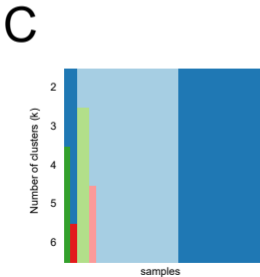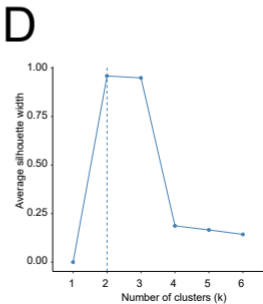

Supplement: Supplementary 1 — Supplementary Figure 1: consensus clustering for NPC samples from GSE12452. (a) Consensus matrix heat map plots when k = 2. (b) The relative change in area under CDF curve for each k subtype. (c) In the tracking plot, the percentages of subtypes were indicated by different colors. (d) Silhouette width of each subtype in NPC. The best subtype number was should be the k value with the highest value of average silhouette width. [file 2242577.f1.pdf]

**A**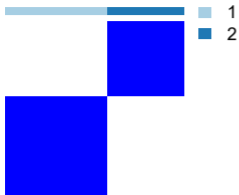**B**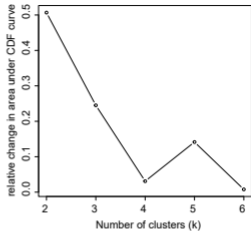**C**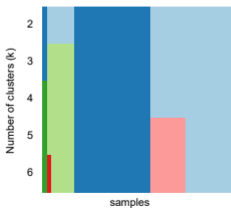**D**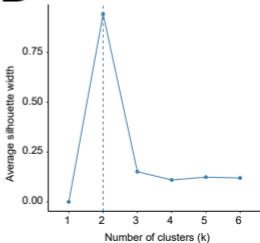

Supplement: Supplementary 3 — Supplementary Figure 3: consensus clustering for NPC samples from GSE68799. (a) Consensus matrix heat map plots when k = 2. (b) The relative change in area under CDF curve for each k subtype. (c) In the tracking plot, the percentages of subtypes were indicated by different colors. (d) Silhouette width of each subtype in NPC. The best subtype number was should be the k value with the highest value of average silhouette width. [file 2242577.f3.pdf]

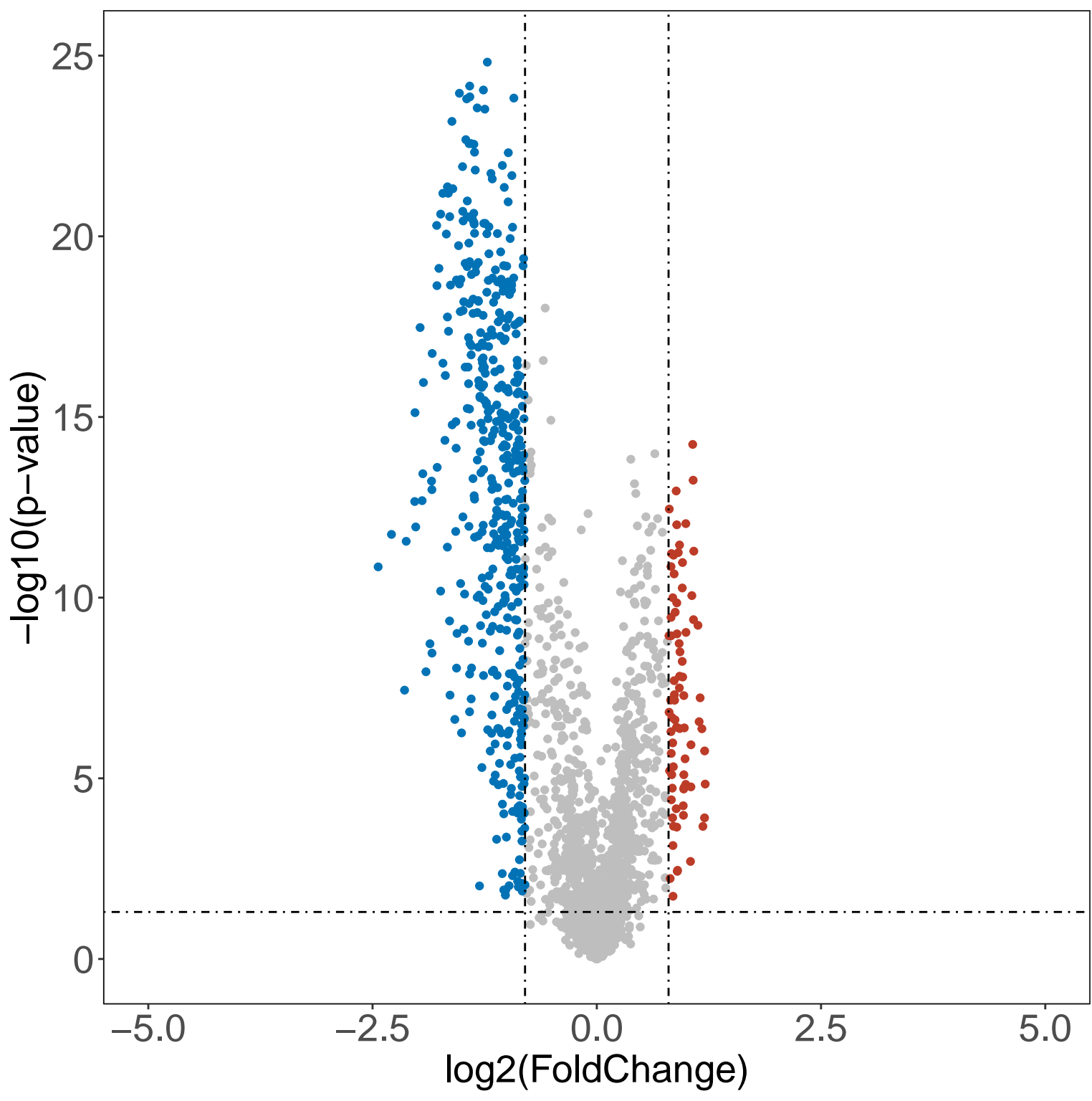

Supplement: Supplementary 5 — Supplementary Figure 5: volcano plot showing the gene expression differences between NPC subtypes. Blue dots, downregulated genes in S2. Red dots, upregulated genes in S2. [file 2242577.f5.pdf]

# Sample clustering to detect outliers

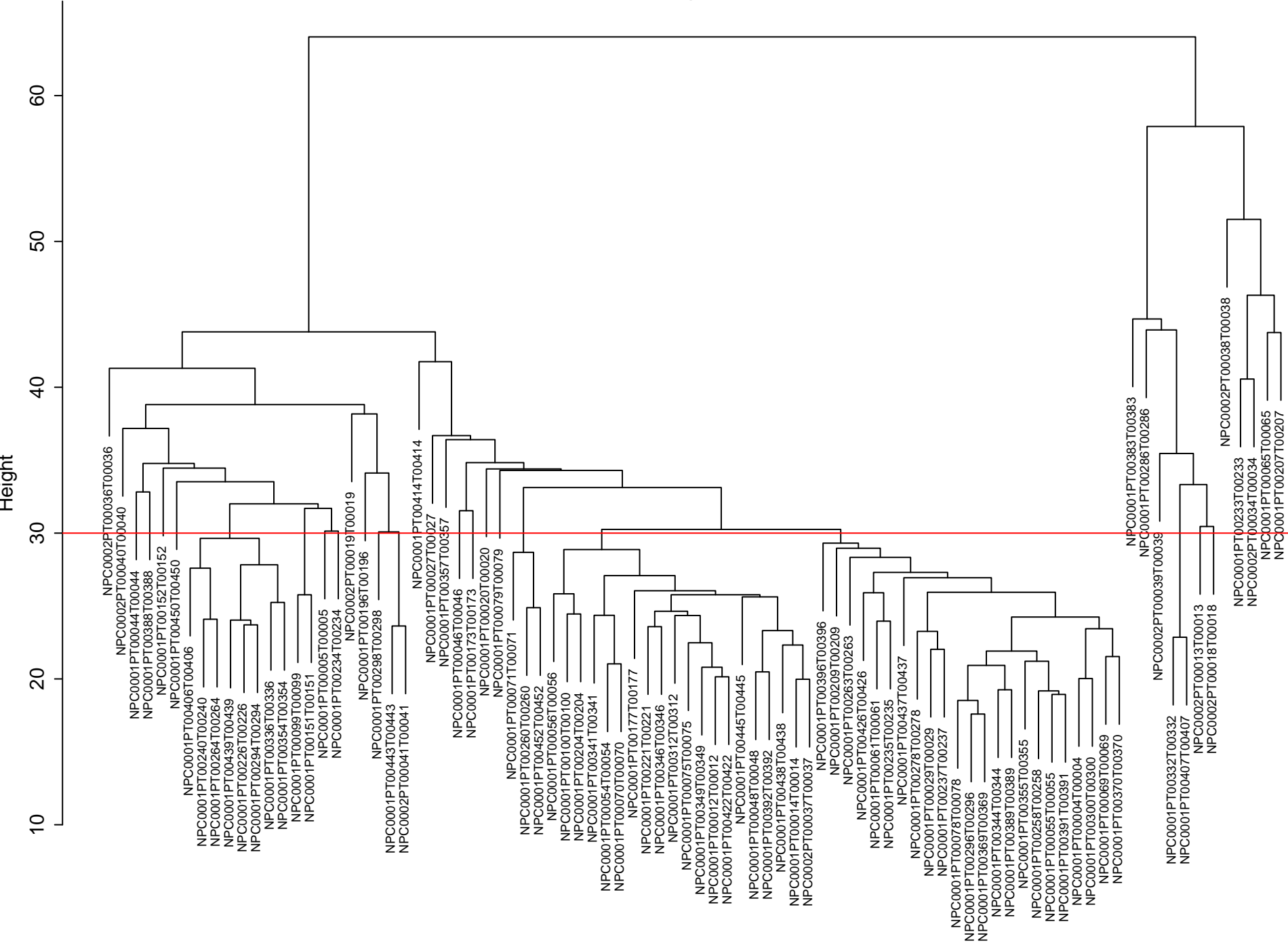

Supplement: Supplementary 6 — Supplementary Figure 6: clustering of samples and removal of outliers. [file 2242577.f6.pdf]
