# Supplementary material for: Nasopharyngeal Carcinoma Subtype Discovery via Immune Cell Scores from Tumor Microenvironment
Source: J Immunol Res. 2023 Mar 31;2023:2242577. doi: 10.1155/2023/2242577 (PMC10234372; doi:10.1155/2023/2242577)
Supplement: Supplementary 2 — Supplementary Figure 2: cell scores in two different NPC subtypes are displayed by heat map (yellow: higher value; blue: lower value). [file 2242577.f2.pdf]

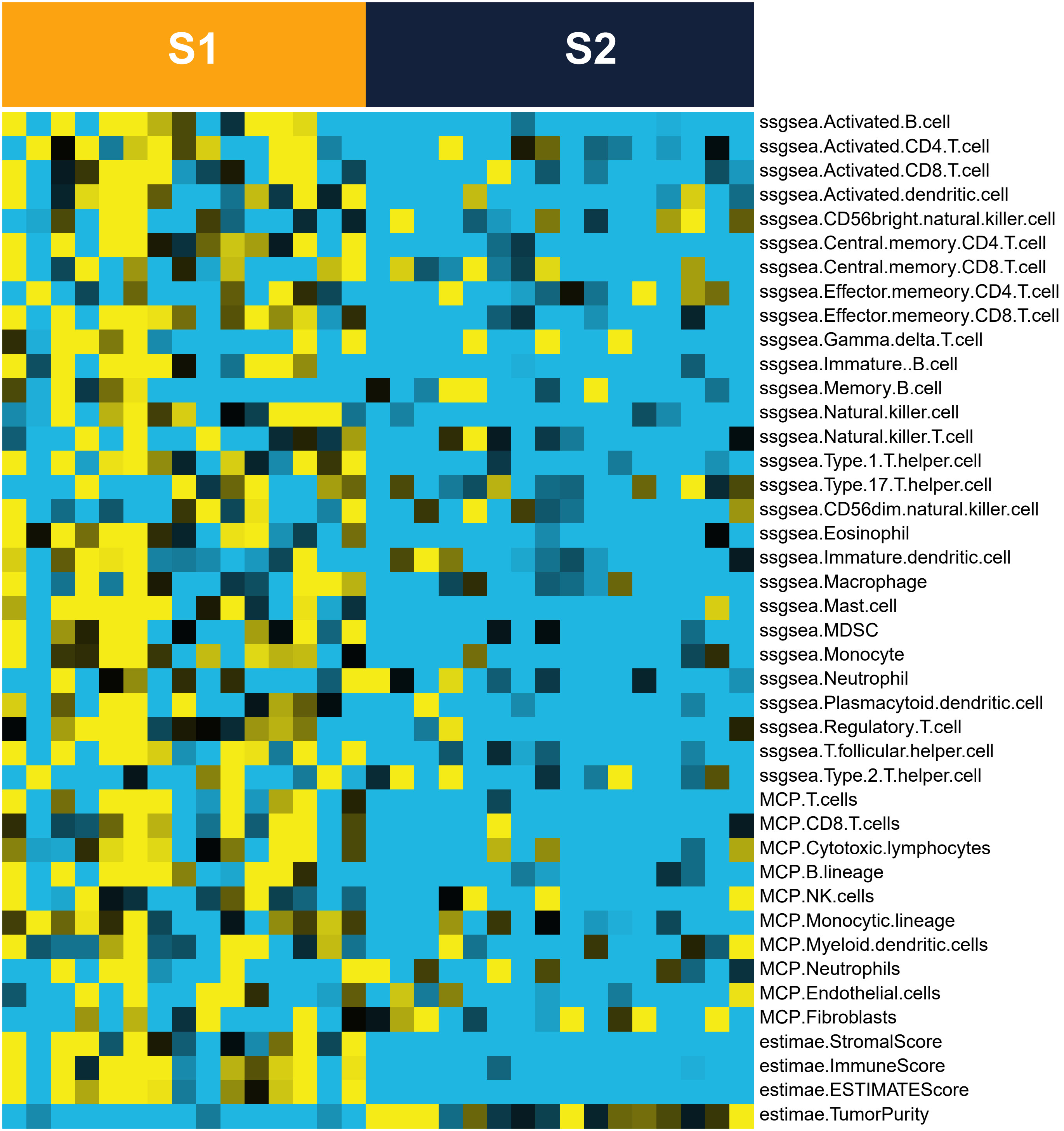
S1
S2
ssgsea.Activated.B.cell
ssgsea.Activated.CD4.T.cell
ssgsea.Activated.CD8.T.cell
ssgsea.Activated.dendritic.cell
ssgsea.CD56bright.natural.killer.cell
ssgsea.Central.memory.CD4.T.cell
ssgsea.Central.memory.CD8.T.cell
ssgsea.Effector.memeory.CD4.T.cell
ssgsea.Effector.memeory.CD8.T.cell
ssgsea.Gamma.delta.T.cell
ssgsea.Immature..B.cell
ssgsea.Memory.B.cell
ssgsea.Natural.killer.cell
ssgsea.Natural.killer.T.cell
ssgsea.Type.1.T.helper.cell
ssgsea.Type.17.T.helper.cell
ssgsea.CD56dim.natural.killer.cell
ssgsea.Eosinophil
ssgsea.Immature.dendritic.cell
ssgsea.Macrophage
ssgsea.Mast.cell
ssgsea.MDSC
ssgsea.Monocyte
ssgsea.Neutrophil
ssgsea.Plasmacytoid.dendritic.cell
ssgsea.Regulatory.T.cell
ssgsea.T.follicular.helper.cell
ssgsea.Type.2.T.helper.cell
MCP.T.cells
MCP.CD8.T.cells
MCP.Cytotoxic.lymphocytes
MCP.B.lineage
MCP.NK.cells
MCP.Monocytic.lineage
MCP.Myeloid.dendritic.cells
MCP.Neutrophils
MCP.Endothelial.cells
MCP.Fibroblasts
estimae.StromalScore
estimae.ImmuneScore
estimae.ESTIMATEScore
estimae.TumorPurity
