# Supplementary material for: Nasopharyngeal Carcinoma Subtype Discovery via Immune Cell Scores from Tumor Microenvironment
Source: J Immunol Res. 2023 Mar 31;2023:2242577. doi: 10.1155/2023/2242577 (PMC10234372; doi:10.1155/2023/2242577)

Step1. Provide the expression values of hub genes

Step2. Click the submit button

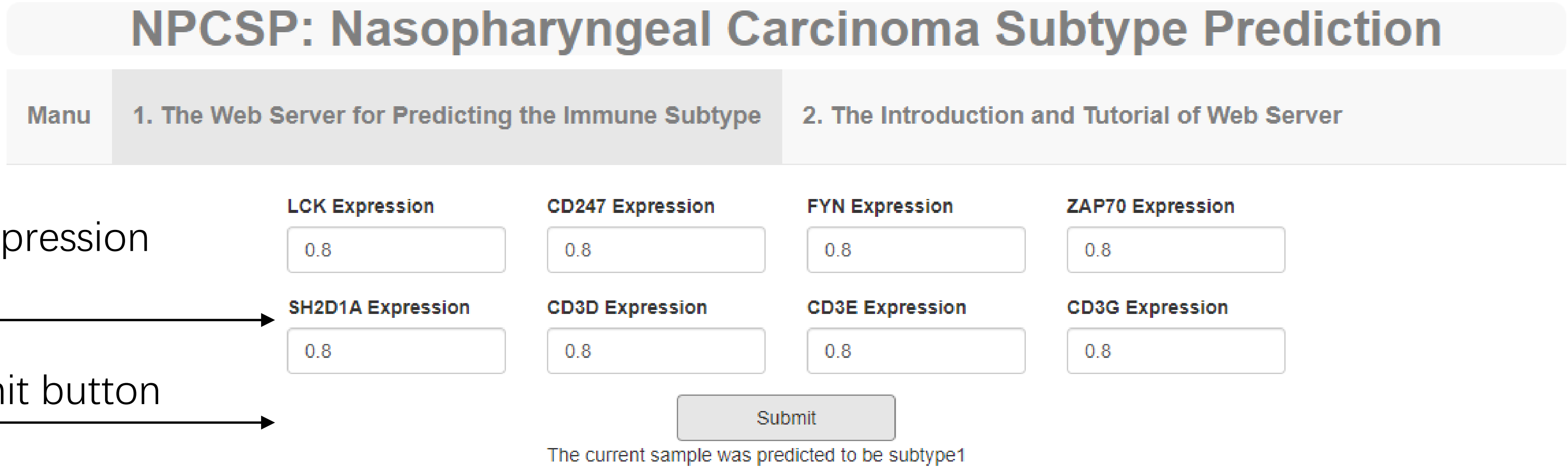

Supplement: Supplementary 7 — Supplementary Figure 7: the tutorial for using the constructed web server. [file 2242577.f7.pdf]
