# Supplementary material for: Nasopharyngeal Carcinoma Subtype Discovery via Immune Cell Scores from Tumor Microenvironment
Source: J Immunol Res. 2023 Mar 31;2023:2242577. doi: 10.1155/2023/2242577 (PMC10234372; doi:10.1155/2023/2242577)
Supplement: Supplementary 8 — Supplementary Table 1: the clinical parameters of datasets (GSE12452, GSE68799, and GSE102349). Supplementary Table 2: associations of NPC subtypes with clinical features in the GSE102349 cohort. Supplementary Table 3: enriched BP items between S1 and S2 subtypes from gene set enrichment analysis. Normalized enrichment score (NES). Positive NES value means that the pathway is enriched in S2 subtype. Supplementary Table 4: enriched MF items between S1 and S2 subtypes from gene set enrichment analysis. Normalized enrichment score (NES). Positive NES value means that the pathway is enriched in S2 subtype. Supplementary Table 5: enriched CC items between S1 and S2 subtypes from gene set enrichment analysis. Normalized enrichment score (NES). Positive NES value means that the pathway is enriched in S2 subtype. Supplementary Table 6: enriched KEGG pathways between S1 and S2 subtypes from gene set enrichment analysis. Normalized enrichment score (NES). Positive NES value means that the pathway is enriched in S2 subtype. Supplementary Table 7: enriched Reactome items between S1 and S2 subtypes from gene set enrichment analysis. Normalized enrichment score (NES). Positive NES value means that the pathway is enriched in S2 subtype. [file 2242577.f8.docx]

| GSE12452 (n=31) | GSE68799 (n=42) | GSE102349 (n=113) |
| --- | --- | --- |
| T1 (n=16)  T2 (n=11)  T3 (n=4) | female (n=9)  Male (n=33) | Stage I (n=5)  Stage II (n=2)  Stage III (n=41)  Stage IV (n=25)  Not available (n=40) |
| N0 (n=7)  N1 (n=16)  N2 (n=8) | Stage II (n=6)  Stage III (n=36) |  |

**Supplementary Table 1.** The clinical parameters of datasets (GSE12452, GSE68799, and GSE102349).

|  | S1 (n=56) | S2 (n=57) | p-value |
| --- | --- | --- | --- |
| Stage  stage1  Stage2  Stage3  Stage4 | 4 (11.8%)  1 (2.94%)  19 (55.9%)  10 (29.4%) | 1 (2.56%)  1 (2.56%)  22 (56.4%)  15 (38.5%) | 0.467 |
| Mean (TMB) | 157 | 129 | 0.310 |

**Supplementary Table 2.** Associations of NPC subtypes with clinical features in the GSE102349 cohort.

| pathway | p-value | NES | size |
| --- | --- | --- | --- |
| BP CARDIAC CHAMBER DEVELOPMENT | 0.023 | 1.33 | 142 |
| BP DNA DEPENDENT DNA REPLICATION MAINTENANCE OF FIDELITY | 0.005 | 1.92 | 22 |
| BP CIRCADIAN RHYTHM | 0.044 | 1.23 | 136 |
| BP SPINAL CORD DEVELOPMENT | 0.009 | 1.56 | 105 |
| BP CELLULAR RESPONSE TO LIPOPROTEIN PARTICLE STIMULUS | 0.033 | -1.50 | 13 |
| BP POSITIVE REGULATION OF KINASE ACTIVITY | 0.004 | -1.40 | 472 |
| BP REGULATION OF T CELL RECEPTOR SIGNALING PATHWAY | 0.002 | -1.89 | 27 |
| BP MOVEMENT IN ENVIRONMENT OF OTHER ORGANISM INVOLVED IN SYMBIOTIC INTERACTION | 0.007 | -1.54 | 85 |
| BP REGULATION OF ESTABLISHMENT OF PLANAR POLARITY | 0.010 | 1.47 | 110 |
| BP MITOTIC CYTOKINESIS | 0.006 | 1.58 | 31 |
| BP NEGATIVE REGULATION OF RESPONSE TO ENDOPLASMIC RETICULUM STRESS | 0.026 | -1.5 | 37 |
| BP CYTOPLASMIC TRANSLATION | 0.005 | 2.06 | 40 |
| BP POSITIVE REGULATION OF CALCIUM ION TRANSPORT | 0.002 | -1.78 | 106 |
| BP REGULATION OF DOUBLE STRAND BREAK REPAIR | 0.005 | 1.69 | 37 |
| BP REGULATION OF B CELL RECEPTOR SIGNALING PATHWAY | 0.003 | -1.90 | 13 |
| BP REGULATION OF G PROTEIN COUPLED RECEPTOR PROTEIN SIGNALING PATHWAY | 0.004 | -1.57 | 125 |
| BP DENDRITE DEVELOPMENT | 0.008 | 1.48 | 79 |
| BP POSITIVE REGULATION OF MYOTUBE DIFFERENTIATION | 0.002 | -1.73 | 29 |
| BP REGULATION OF CELL ACTIVATION | 0.002 | -2.3 | 459 |
| BP ATP DEPENDENT CHROMATIN REMODELING | 0.007 | 2.08 | 72 |

**Supplementary Table 3.** Enriched BP items between S1 and S2 subtypes from Gene set enrichment analysis. Normalized enrichment score (NES). Positive NES value means that the pathway is enriched in S2 subtype.

| pathway | p-value | NES | size |
| --- | --- | --- | --- |
| MF MANNOSYLTRANSFERASE ACTIVITY | 0.026 | 1.50 | 24 |
| MF TRNA BINDING | 0.044 | 1.30 | 57 |
| MF DNA SECONDARY STRUCTURE BINDING | 0.022 | 1.56 | 35 |
| MF MAGNESIUM ION BINDING | 0.014 | 1.25 | 208 |
| MF FOUR WAY JUNCTION DNA BINDING | 0.026 | 1.56 | 17 |
| MF BASAL TRANSCRIPTION MACHINERY BINDING | 0.038 | 1.33 | 58 |
| MF LIPOPOLYSACCHARIDE BINDING | 0.006 | -1.74 | 29 |
| MF AMYLOID BETA BINDING | 0.002 | -1.74 | 85 |
| MF G PROTEIN COUPLED CHEMOATTRACTANT RECEPTOR ACTIVITY | 0.003 | -1.95 | 23 |
| MF PEPTIDE RECEPTOR ACTIVITY | 0.002 | -1.89 | 146 |
| MF G PROTEIN COUPLED RECEPTOR BINDING | 0.002 | -1.55 | 267 |
| MF PHOSPHOTYROSINE RESIDUE BINDING | 0.009 | -1.67 | 44 |
| MF PHOSPHATIDYLSERINE BINDING | 0.005 | -1.58 | 59 |
| MF COMPLEMENT BINDING | 0.006 | -1.73 | 19 |
| MF LIPOPOLYSACCHARIDE IMMUNE RECEPTOR ACTIVITY | 0.003 | -1.56 | 5 |
| MF FIBRONECTIN BINDING | 0.019 | -1.57 | 29 |
| MF PROTEASE BINDING | 0.002 | -1.61 | 131 |
| MF P53 BINDING | 0.040 | 1.31 | 64 |
| MF DNA HELICASE ACTIVITY | 0.007 | 2.27 | 70 |
| MF DAMAGED DNA BINDING | 0.007 | 1.83 | 67 |

**Supplementary Table 4.** Enriched MF items between S1 and S2 subtypes from Gene set enrichment analysis. Normalized enrichment score (NES). Positive NES value means that the pathway is enriched in S2 subtype.

| pathway | p-value | NES | Size |
| --- | --- | --- | --- |
| CC HISTONE DEACETYLASE COMPLEX | 0.008 | 1.80 | 70 |
| CC UBIQUITIN LIGASE COMPLEX | 0.033 | 1.32 | 286 |
| CC NUCLEAR UBIQUITIN LIGASE COMPLEX | 0.023 | 1.64 | 43 |
| CC CYTOPLASMIC UBIQUITIN LIGASE COMPLEX | 0.046 | -1.47 | 12 |
| CC NUCLEAR CHROMOSOME | 0.017 | 2.14 | 188 |
| CC CYCLIN DEPENDENT PROTEIN KINASE HOLOENZYME COMPLEX | 0.032 | 1.56 | 46 |
| CC ORGANELLAR RIBOSOME | 0.009 | 2.23 | 78 |
| CC TRANSCRIPTION EXPORT COMPLEX | 0.028 | 1.58 | 10 |
| CC PROTEASOME COMPLEX | 0.034 | 1.40 | 58 |
| CC CHROMOSOME CENTROMERIC REGION | 0.016 | 2.13 | 193 |
| CC CONDENSED CHROMOSOME CENTROMERIC REGION | 0.012 | 2.27 | 141 |
| CC CHROMOSOME TELOMERIC REGION | 0.012 | 1.81 | 136 |
| CC HETEROCHROMATIN | 0.008 | 1.82 | 61 |
| CC CONDENSED CHROMOSOME | 0.018 | 2.15 | 230 |
| CC CONDENSED NUCLEAR CHROMOSOME | 0.008 | 1.55 | 65 |
| CC CONDENSIN COMPLEX | 0.042 | 1.55 | 8 |
| CC ORIGIN RECOGNITION COMPLEX | 0.023 | 1.66 | 9 |
| CC SPINDLE POLE | 0.013 | 1.89 | 160 |
| CC OUTER KINETOCHORE | 0.010 | 1.80 | 11 |
| CC CORNIFIED ENVELOPE | 0.039 | 1.50 | 44 |

**Supplementary Table 5.** Enriched CC items between S1 and S2 subtypes from Gene set enrichment analysis. Normalized enrichment score (NES). Positive NES value means that the pathway is enriched in S2 subtype.

| pathway | p-value | NES | size |
| --- | --- | --- | --- |
| KEGG OXIDATIVE PHOSPHORYLATION | 0.043 | 1.36 | 116 |
| KEGG PYRIMIDINE METABOLISM | 0.008 | 1.58 | 96 |
| KEGG LYSINE DEGRADATION | 0.012 | 1.55 | 44 |
| KEGG TRYPTOPHAN METABOLISM | 0.020 | -1.48 | 40 |
| KEGG OTHER GLYCAN DEGRADATION | 0.003 | -1.71 | 16 |
| KEGG GLYCOSAMINOGLYCAN DEGRADATION | 0.047 | -1.44 | 21 |
| KEGG ARACHIDONIC ACID METABOLISM | 0.014 | -1.53 | 58 |
| KEGG GLYCOSPHINGOLIPID BIOSYNTHESIS GLOBO SERIES | 0.046 | -1.41 | 14 |
| KEGG GLYCOSPHINGOLIPID BIOSYNTHESIS GANGLIO SERIES | 0.003 | -1.66 | 15 |
| KEGG NICOTINATE AND NICOTINAMIDE METABOLISM | 0.031 | -1.47 | 22 |
| KEGG AMINOACYL TRNA BIOSYNTHESIS | 0.006 | 1.67 | 41 |
| KEGG DRUG METABOLISM CYTOCHROME P450 | 0.016 | -1.47 | 72 |
| KEGG RIBOSOME | 0.007 | 2.12 | 86 |
| KEGG RNA DEGRADATION | 0.046 | 1.34 | 57 |
| KEGG RNA POLYMERASE | 0.011 | 1.65 | 29 |
| KEGG BASAL TRANSCRIPTION FACTORS | 0.006 | 1.65 | 35 |
| KEGG DNA REPLICATION | 0.006 | 1.92 | 36 |
| KEGG SPLICEOSOME | 0.010 | 2.16 | 125 |
| KEGG NUCLEOTIDE EXCISION REPAIR | 0.006 | 1.57 | 44 |
| KEGG MISMATCH REPAIR | 0.011 | 1.69 | 23 |

**Supplementary Table 6.** Enriched KEGG pathways between S1 and S2 subtypes from Gene set enrichment analysis. Normalized enrichment score (NES). Positive NES value means that the pathway is enriched in S2 subtype.

| pathway | p-value | NES | size |
| --- | --- | --- | --- |
| REACTOME TRANSLATION | 0.014 | 2.03 | 144 |
| REACTOME RNA POL III TRANSCRIPTION INITIATION FROM TYPE 2 PROMOTER | 0.005 | 1.66 | 23 |
| REACTOME RNA POL I TRANSCRIPTION TERMINATION | 0.005 | 1.72 | 21 |
| REACTOME FORMATION OF THE TERNARY COMPLEX AND SUBSEQUENTLY THE 43S COMPLEX | 0.007 | 1.79 | 48 |
| REACTOME ACTIVATION OF THE PRE REPLICATIVE COMPLEX | 0.005 | 2.14 | 30 |
| REACTOME SIGNALING BY RHO GTPASES | 0.007 | -1.52 | 112 |
| REACTOME METABOLISM OF NON CODING RNA | 0.007 | 2.02 | 48 |
| REACTOME SIGNALING BY NODAL | 0.017 | 1.62 | 18 |
| REACTOME TCA CYCLE AND RESPIRATORY ELECTRON TRANSPORT | 0.012 | 1.82 | 117 |
| REACTOME ANTIGEN PROCESSING CROSS PRESENTATION | 0.023 | -1.48 | 72 |
| REACTOME TRIF MEDIATED TLR3 SIGNALING | 0.021 | -1.50 | 72 |
| REACTOME ENDOSOMAL VACUOLAR PATHWAY | 0.003 | -1.68 | 8 |
| REACTOME G0 AND EARLY G1 | 0.005 | 1.81 | 23 |
| REACTOME NEF MEDIATES DOWN MODULATION OF CELL SURFACE RECEPTORS BY RECRUITING THEM TO CLATHRIN ADAPTERS | 0.006 | -1.71 | 20 |
| REACTOME IMMUNOREGULATORY INTERACTIONS BETWEEN A LYMPHOID AND A NON LYMPHOID CELL | 0.002 | -2.32 | 61 |
| REACTOME IL 7 SIGNALING | 0.014 | -1.55 | 11 |
| REACTOME CELL CYCLE | 0.043 | 2.16 | 395 |
| REACTOME ORC1 REMOVAL FROM CHROMATIN | 0.008 | 1.76 | 65 |
| REACTOME DEFENSINS | 0.003 | -1.87 | 22 |
| REACTOME BETA DEFENSINS | 0.003 | -1.84 | 15 |

**Supplementary Table 7.** Enriched REACTOME items between S1 and S2 subtypes from Gene set enrichment analysis. Normalized enrichment score (NES). Positive NES value means that the pathway is enriched in S2 subtype.
